# Male characteristics and contraception in four districts of the central region, Ghana

**DOI:** 10.1186/s40834-023-00245-9

**Published:** 2023-08-25

**Authors:** Terence A. Longla, Deda Ogum-Alangea, Adolphina Addo-Lartey, Adom A. Manu, Richard M.K. Adanu

**Affiliations:** 1 Department of Population, Family and Reproductive Health, University of Ghana School of Public Health, Legon, Ghana; 2 Department of Epidemiology and Disease Control, University of Ghana School of Public Health, Legon, Ghana

**Keywords:** Male, Characteristics, Contraception, Ghana

## Abstract

**Background:**

A lack of male involvement in contraception can negatively affect its practice. To promote male participation in family planning, there is a dire need to understand male attributes that play a role in contraception. This study focuses on the male characteristics that influence the practice of traditional and modern methods of contraception.

**Methods:**

This study is a secondary analysis of quantitative data obtained from the baseline assessment of the Ghana Community-Based Action Teams Study that aimed to prevent violence against women in the Central Region of Ghana in 2016. The analysis included 1742 partnered males aged 18–60 years. Chi-square test, t-test and logistic regression analyses were used to assess the association between male characteristics and the practice of contraception (significance level = 0.05).

**Results:**

The prevalence of contraception was 24.4% (95% CI = 20.8–28.5). Significant male characteristics that were positively associated with the practice of contraception in adjusted models were: post-primary education (AOR = 1.96, 95% CI = 1.27–3.04), perpetration of Intimate Partner Violence (AOR = 1.83, 95% CI = 1.49–2.26), and the number of main sexual partners (AOR = 1.78, 95% CI = 1.15–2.75). However, wanting the first child (AOR = 0.71, 95% CI = 0.54–0.94) and male controlling behaviour (AOR = 0.7, 95% CI = 0.49–0.99) statistically significantly associated with reduced odds of practicing contraception.

**Conclusion:**

Male partner characteristics influence the practice of contraception. Family planning sensitization and education programs should target males who are less likely to practice contraception.

## Background

Family planning enables couples to have an optimal number of children and decide on pregnancy spacing. It is accomplished by knowledge, education, and the use of methods of contraception [1]. Contraception can broadly be classified into traditional and modern forms, with few dependent on males; condoms, withdrawal method, rhythm method and vasectomy [2]. Contraception has a couple of advantages, including a reduction in maternal and infant mortalities [3], reduction of adolescent pregnancy [3], prevention of sexually transmitted infections [4] and controlling population growth, amongst others. Their use is often affected by individual and couple-level factors.

Often, there is a lack of male involvement in contraception. This can have serious consequences, especially in a developing country like Ghana, where they constitute the majority of the household and nation’s decision-making authority [5]. There is evidence that having good knowledge of contraception is generally considered a vital step toward its uptake [6–8]. The current evidence based on the 2014 Ghana DHS data shows an almost universal knowledge of one or more contraceptive methods among adult populations [9]. Despite this high level of awareness on this subject, and the availability of low-cost family planning services, its uptake in most households in Ghana is paradoxically very low. According to the Ghana DHS data in 2014, the prevalence of contraception among married women of reproductive age was 26.7% (22.2% for modern methods).

Furthermore, evidence from a maternal health survey showed that only one-third (31%) of married women in the reproductive age group used contraception: 25% used modern, while 6% practiced traditional methods of contraception [10]. This prevalence is very low compared to a global modern contraceptive prevalence rate of 57% [11]. Globally, the prevalence of male methods of contraception in 2017 was 15.7%, while that of Ghana was 5.5% [2]. These findings highlight the low utilization of contraception in Ghana. In 2017, a study conducted in Ghana showed that only 7.2% of men accompanied their spouses to the clinic to seek family planning services [12]. This suggests poor male involvement in contraception, which can have implications for uptake. About 30% of Ghanaians have an unmet need for contraception [9], which will inevitably result in adverse outcomes. Globally, about 20 million cases of unsafe abortions are noted every year, with resultant 67,000 deaths, most of which occur in underdeveloped countries [11]. Ameyaw et al. (2019), reported, after an analysis of the 2014 Demographic and Health Survey Data, an unwanted pregnancy rate of 28.9% in Ghana [13]. An increased rate of unwanted pregnancies within households may result in abortion, most of which are unsafe in our settings, and this may impose severe consequences on the reproductive health of the people concerned [11, 14].

Even though some studies have explored male factors that influence contraceptive use, most of these studies involve female respondents. The relatively little attention paid to males, who make most of the household decisions in our milieu, can negatively impact the use of family planning services. This study sought to determine the prevalence and predictors of contraception (traditional and modern methods) among men in the Central Region of Ghana. An analysis of the male characteristics that influence the practice of contraception can provide evidence that will be used to improve male involvement in family planning programs.

## Methods

### Study design

This study is a secondary analysis of quantitative data obtained from the baseline assessment of a community randomized controlled trial in the Central Region of Ghana that ran for three years starting in 2016. The aim of the trial, dubbed “the Ghana Community-based action teams (COMBAT) Health Promotion Study,“ was to evaluate the impact of the Rural Response System (RRS) at the community level to prevent violence against women in Ghana using Community-Based Action Teams [15]. The study was an unmatched cluster randomized controlled trial with two arms that utilized a multistage sampling technique involving rural and urban communities. The intervention arm received the RRS intervention to prevent IPV for 18 months, while the control group did not receive any intervention. Quantitative data were collected at baseline and endpoint of the study, with the use of a well-structured interviewer-administered questionnaire, from women (18–49 years) and men (more than 18 years) who lived in a particular household for at least one year, could communicate in English, Twi or Fante and did not have a mental illness [15].

The current study involves the analysis of the quantitative data collected from partnered males (either married or had a girlfriend) at baseline and excluded those older than 60 years or whose partners were pregnant. Of the 2126 males interviewed at baseline, 1742 were eligible for this study.

### Study area

The study involved four districts that were separated by at least one district to prevent the diffusion of the intervention from the intervention to the control arms. Both intervention and control arms were assigned one coastal and one inland district, representative of the region’s topography. The coastal districts mostly consisted of urban and peri-urban communities while the inland districts had a mix of urban, rural and per-urban communities. The participating districts include Upper Denkyira, Abura/Asebu/Kwamankese, Agona and Komenda/Edina/Eguafo/Abreim of the Central Region of Ghana. Ghana is located in West Africa with 27 million inhabitants (Population and Housing Census, 2010), and now has [16] administrative regions. The Central region has a total population of 2,413,050 (2013). About half of the adult inhabitants are literate, with men contributing to 69.8% of this proportion.

### Study variables

The outcome variable is the practice of male-reported contraception between couples. The men were asked the question, “Are you and your partner (girlfriend/wife) doing anything to avoid pregnancy? “An affirmative answer to this question was considered as the practice of contraception, provided the reported method of contraception is a recognized and documented modern or traditional method.

The predictor variables considered in this analysis cut across socio-demographic, socio-cultural, behavioral, and sexual/reproductive factors.

Eight socio-demographic variables were assessed to determine their association with the outcome variable. The respondent’s age and the age at which they first got married were recorded and used as continuous variables. The age difference with their partners, age at first birth, marital status, area of residence, income level, and education level were used as categorical variables.

The prevailing social norms, individual gender attitudes, controlling behaviour, perpetration of intimate partner violence (IPV), depression, substance use, decision-making authority and life satisfaction are the socio-cultural and behavioral factors that were considered in the study. The community’s prevailing social norms concern what the community thinks about the relations between males and females. Male controlling behavior was defined as a man taking charge of and determining his partner’s actions [17]. Intimate Partner Violence was defined as any form of sexual, physical, emotional or economic violence perpetrated by the man against his partner within the past 12 months. In this study, the prevailing social norms, individual gender attitudes, controlling behavior, IPV and life satisfaction were assessed on a Likert scale containing some items (questions) using appropriate tools [15, 18]. Additive scores were computed for each of these variables from this scale after Cronbach alpha was used to check for internal consistency [15, 18]. The scores for gender attitudes, controlling behavior and IPV were categorized into two, while social norms and life satisfaction were measured on a continuous scale in this current analysis. Depression was assessed using the Center for Epidemiological Studies Depression (CES-D) scale for research. A composite score was generated (Cronbach’s alpha coefficient = 0.86), with a high score indicating a higher probability of being depressed [15]. To assess substance use, they were asked if they had taken either alcohol or drugs in the past 12 months. The details of how these variables are measured are reported elsewhere [15, 18].

Finally, the sexual/reproductive variables considered in this study were the number of biological children, main sexual partners, other sexual partners and whether they wanted their first child at birth (for those who had a child). The number of biological children was recorded as a continuous variable while the others were categorized.

### Data analysis

Data analysis was done using STATA version 16. The variables of interest were identified from the primary data set and kept with unique identifiers in a separate STATA file. The data was cleaned by running frequencies of the various variables to identify and label odd or missing entries. The variables of interest were recoded to obtain desired measures, and new variables and categories were created by transforming certain desired variables and saved for analysis. The complex nature of the survey, i.e., stratification and clustering, was considered during the analysis to obtain sample estimates that represent the districts involved.

Descriptive statistics were computed for the various variables: mean and standard deviation were calculated for continuous variables, while proportions were calculated for categorical variables. Univariable analysis was done using the Chi-square test, students t-test and simple logistic regression (to measure crude associations between the outcome and predictor variables). Variables that were significantly associated with the practice of contraception in univariable analysis, together with some key socio-demographic characteristics, were moved to multivariable regression models (multivariable analysis) to determine the predictors of contraception. The level of statistical significance was set at 5% (p-value of 0.05). Statistical analysis was based on complete cases since less than 1% of the data was missing.

### Ethical considerations

The candidate obtained permission from the primary investigators of the Ghana COMBAT study to use the de-identified data set for the current analysis. The primary investigators obtained ethical clearance from the Noguchi Memorial Institute for Medical Research Institutional Ethics Board (NMIMR-IRB), Legon, University of Ghana (number 006/15–16) and the South African Medical Research Council (EC031-9/2015).

## Results

### Socio-demographic characteristics of the participants

The socio-demographic characteristics of the respondents are summarized in Table 1 below. The study participants’ age ranged from 18 to 60 years, with a mean of 36.0 ± 11.3 years. Averagely, they first got married at the age of 25.8 (SD = 5.3) years. Among those with at least a child, a greater majority (93.2%) had their first child after 19 years. Most (62.8%) of the men were married, while the rest had at least one sexual partner. Up to 29.7% of the men did not earn any income, and only a very small proportion earned more than 1000 cedis during the month preceding the survey. Regarding education, up to 17.7% did not have formal education, while one-third (33.3%) attended at least junior secondary school.

### Other characteristics of the respondents

Half (50.0%) of the men had equitable gender attitudes, and 47.1% reported controlling behaviour. Most of the participants (80.1%) believe that the man should be the one to make the household’s final decisions. Slightly more than one-third (37.3%) of them reported that they had perpetrated at least one form of IPV, with the most common single forms being sexual IPV (17.5%) and physical IPV (12.8%). Less than one-third of the men (27.8%) did not want to have their first child when they had them. About three-quarters of the men (74.7%) had one main sexual partner. These characteristics are summarized in Table 2.

### Prevalence of contraception

Of the 1742 males, 425 practiced contraception, i.e., the prevalence was 24.4% (95% CI:20.8–28.5).

### Predictors of the practice of contraception

#### Univariable analysis

The association between the socio-demographic characteristics of the males and the practice of contraception is summarized in Table 1. The average age of those who practiced contraception was significantly lower than that of those who did not (34.4 ± 10.3 vs. 36.8 ± 11.5, p-value < 0.001). A lesser proportion of married men practiced contraception compared to their unmarried counterparts (21.5% vs. 29.3%, p-value = 0.001). Males with a higher income level were more likely to practice contraception than those without income (p-value < 0.001). Formally educated males were more likely to practice contraception compared to those who did not attend school (p-value < 0.001). More men in inland areas practiced contraception compared to the coastal dwellers (29.2% vs. 20.3%, p-value = 0.006).

Other behavioral and sexual/reproductive characteristics of the males were also associated with the practice of contraception (Table 2). Favorable social norm was associated with a higher likelihood of practicing contraception. The mean social norm score of the men who practiced contraception was significantly lower than that for those who did not (32.5 ± 6.3 vs. 34.2 ± 6.4, p-value < 0.001). More men with gender-equitable norms practiced contraception compared to their counterparts with gender-inequitable norms (20.3% vs. 28.5%, p-value = 0.001). Participants who believed that males should be the ones to make the final decisions in the household were less likely to practice contraception compared to those who thought otherwise (22.0% vs. 34%, p-value < 0.001). A significantly higher percentage of men who had sexual partners other than their main sexual partners practiced contraception compared to those who did not have other sexual partners (29.1% vs. 18.6%, p-value = 0.002). Men involved in transactional sex were more likely to practice contraception than those who did not indulge in transactional sex (30.4% vs. 22.2%, p-value = 0.001). Controlling behavior, perpetration of IPV, number of main sexual partners and whether the first child was wanted were also associated with the practice of contraception.

**Table 1:** Male Socio-demographic characteristics and the practice of contraception

Respondent characteristics	Total (%)	Use of contraception			p-value	
		**Yes**	**No**			
		**N (%)/Mean ± SD**		**N (%)/Mean ± SD**		
Age (years)		34.4 ± 10.3		36.8 ± 11.5		< 0.001*
Age at first marriage (years)		26.1 ± 4.8		25.8 ± 5.4		0.494*
Age difference with partner						
Partner ≥ 5 years younger Partner < 5 years younger	792(47.6) 871(52.4)	192(24.2) 217(24.8)		600(75.8) 654(75.2)		0.807
Age at first birth						
< 20 years ≥ 20 years	88(6.8) 1205(93.2)	17(19.3) 276(22.9)		71(80.7) 929(77.1)		0.522
Marital status						
Married Not married	1094(62.8) 648(37.2)	235(21.5) 190(29.3)		859(78.5) 458(70.7)		**0.001**
Income level (GHc)						
Nothing ≤ 200 201–1000 > 1000	469(29.7) 460(29.2) 527(33.4) 121(7.7)	96(20.5) 84(18.3) 151(28.7) 51(42.1)		373(79.5) 376(81.7) 376(71.3) 70(57.94)		0.389 **< 0.001** **< 0.001**
Educational level						
None Primary Junior secondary Senior secondary Post-secondary	309(17.7) 296(16.9) 581(33.3) 352(20.5) 204(11.6)	32(10.4) 53(17.9) 151(26) 110(31.2) 79(38.7)		277(89.6) 243(82.1) 430(74.0) 242(68.8) 125(61.3)		**< 0.001** **< 0.001** **< 0.001** **< 0.001**
Area of residence						
Coastal Inland	940(54.0) 802(46.0)	191(20.3) 234(29.2)		749(79.7) 568(70.8)		**0.006**

*p-value from t-test, others from Chi-square tests

**Table 2:** Other male characteristics and the practice of contraception

Respondent characteristics	Total (%)	Use of contraception			p-value
		**Yes**		**No**	
		**N (%)/Mean ± SD**	**N (%)/Mean ± SD**		
Social norms score		32.5 ± 6.3	34.2(6.4)		< 0.001*
Individual gender attitudes					
Equitable	871(50.0)	248(28.5)	623(71.5)		
Inequitable	871(50.0)	177(20.3)	694(79.7)		**0.001**
Controlling behavior					
No	921(52.9)	273(29.6)	648(70.4)		
Yes	821(47.1)	152(18.5)	669(81.5)		**< 0.001**
Decision-making authority					
Not man	347(19.9)	118(34.0)	229(66.0)		
Man	1395(80.1)	307(22.0)	1088(78.0)		**< 0.001**
Perpetrate IPV					
No	1093(62.7)	220(20.1)	873(79.9)		
Yes	649(37.3)	205(31.6)	444(68.4)		**< 0.001**
Life satisfaction score		14.7 ± 4.0	15.1 ± 4.2		0.093*
Depression score		35.8 ± 9.2	36.6 ± 9.3		0.108*
Substance use					
No	958(55.0)	241(25.2)	717(74.8)		0.428
Yes	783(45.0)	184(23.5)	599(76.5)		
Number of children		3.57 ± 2.25	3.60 ± 2.17		0.878
No. of main sexual partners					
One	1301(74.7)	333(25.6)	968(74.4)		
None	181(10.4)	15(8.3)	166(91.7)		**< 0.001**
More than one	259(14.9)	77(29.7)	182(70.3)		**< 0.001**
Have other sexual partners					
No	1315(75.7)	298(18.6)	1017(81.4)		**0.002**
Yes	422(24.3)	123(29.1)	299(70.9)		
Transactional sex					
No	1275(73.2)	283(22.2)	992(77.8)		
Yes	467(26.8)	142(30.4)	325(69.6)		**0.001**
Wanted first child					
No	360(27.8)	103 (28.6)	257 (71.4)		
Yes	934(72.2)	190 (20.3)	744 (79.7)		**< 0.001**

*p-value from t-test, others from Chi-square test

#### Multivariate analysis (multiple logistic regression)

After controlling for age, marital status, income level, area of residence, social norms, individual gender attitudes, decision-making authority, having other sexual partners and the practice of transactional sex, five male characteristics emerged as significant predictors of contraception. They include the educational level, the number of main sexual partners, perpetration of IPV, controlling behaviour and whether the first child was wanted. There was a statistically significant relationship between those who had post-primary school education and the practice of contraception. For instance, men whose highest level of study was junior secondary school had a 96% increased odds of practicing contraception compared to those with no education (AOR = 1.96, 95% CI: 1.27–3.04, p-value = 0.003). The odds of practicing contraception increased with higher levels of education, as illustrated in Table 3.

Men who reported controlling behaviour had a 30% reduction in the odds of practicing contraception compared to those who did not (AOR = 0.70, 95% CI: 0.49–0.99, p-value = 0.045). Also, males who perpetrated any form of IPV in the last 12 months had 51% higher odds of practicing contraception than those who did not (AOR = 1.51, 95% CI: 1.08–2.13, p-value = 0.018). Men who did not have a main sexual partner had 66% reduced odds of practicing contraception compared to those with one main sexual partner (AOR = 0.34, 95% CI: 0.15–0.75, p-value = 0.009). Furthermore, those with more than one main sexual partner had 78% higher odds of practicing contraception than those with one main sexual partner (AOR = 1.78, 95% CI: 1.15–2.75, p-value = 0.011). Lastly, participants who wanted their first child at the time of delivery had a 29% reduction in the odds of practicing contraception compared to those who did not want their first child at delivery (AOR = 0.71, 95% CI: 0.54–0.94, p-value = 0.018).

**Table 3:** Results of multiple logistic regression (multivariable analysis)

Respondents’ characteristic	Simple logistic regression UOR (95% CI)	Multiple logistic regression	
		**AOR (95% CI)**	**p-value**
Age (years)	0.98 (0.97–0.99)	0.99(0.98-1.00)	0.093
Marital status			
Married Not married	1 1.52 (1.21–1.91)	1 0.89(0.58–1.35)	0.565
Income level (GHc)			
Nothing ≤ 200 201–1000 > 1000	1 0.87 (0.63–1.21) 1.56 (1.24–1.96) 2.83 (1.89–4.24)	1 0.80(0.52–1.22) 1.23(0.94–1.61) 1.52(0.82–2.81)	0.285 0.125 0.175
Educational level			
None Primary Junior secondary Senior secondary Post-secondary	1 1.89 (1.37–2.60) 3.04 (2.15–4.36) 3.93 (2.57–6.03) 5.47 (3.76–7.96)	1 1.44(0.92–2.25) 1.96(1.27–3.04) 2.03(1.22–3.38) 2.56(1.41–4.66)	0.107 **0.003** **0.007** **0.003**
Area of residence			
Coastal Inland	1 1.62 (1.16–2.25)	1 1.04(0.73–1.49)	0.814
Social norms score	0.96 (0.94–0.98)	0.98(0.95–1.02)	0.301
Individual gender attitudes			
Equitable Inequitable	1 0.64 (0.50–0.82)	1 0.97(0.71–1.33)	0.865
Controlling behavior			
No Yes	1 0.54 (0.42–0.69)	1 0.70(0.49–0.99)	**0.045**
Decision-making authority			
Not man Man	1 0.55 (0.43–0.70)	1 0.72(0.47–1.10)	0.121
Perpetrate IPV			
No Yes	1 1.83 (1.49–2.26)	1 1.51(1.08–2.13)	**0.018**
Num. of main sexual partners			
One None More than one	1 0.26 (0.15–0.45) 1.23 (0.93–1.63)	1 0.34(0.15–0.75) 1.78(1.15–2.75)	**0.009** **0.011**
Have other sexual partners			
No Yes	1 1.40 (1.15–1.72)	1 0.89(0.60–1.34)	0.577
Transactional sex			
No Yes	1 1.53 (1.23–1.93)	1 1.04(0.70–1.54)	0.844
Wanted first child			
No Yes	1 0.64 (0.56–0.81)	1 0.71(0.54–0.94)	**0.018**

## Discussion

This study found a 24.4% prevalence of contraception among men in the Central Region of Ghana. This prevalence is probably less than the actual prevalence for these districts because males are more likely to underreport the use of contraceptives compared to females [19]. This is because some females tend to conceal their use of some methods of contraception from their partners [20].

Level of education emerged as one of the independent predictors of contraception in this study, consistent with other studies [21–23]. The higher odds of practicing contraception among educated males can be explained by the fact that educated people will easily access and comprehend information regarding family planning, understand the benefits of limiting birth or the disadvantages of large family size, and thus practice contraception.

Our finding of higher odds of practicing contraception among males who perpetrated violence against their partners is consistent with other studies. Kidman et al. 2015, for example, after analyzing the DHS data for the Democratic Republic of Congo, found that households where men perpetrated sexual IPV, were more likely to use modern contraceptives than homes where IPV was not common [24]. Furthermore, in Pakistan, women who experienced IPV had higher odds of practicing both modern and traditional methods of contraception compared to those who did not experience IPV [25]. These can be explained by the fact that the partners of males who experience IPV will most likely practice contraception to avoid unwanted pregnancies and thus empower themselves since sexual IPV was the most common form of IPV perpetrated by the males in this current study. Contrary to our research, Maxwell et al. 2018, discovered that in Uganda, the perpetration of IPV resulted in reduced odds of using methods of contraception that require the cooperation of both partners, like condoms [26]. This is due to the inability of the victims of IPV to negotiate the use of such methods (e.g., condoms) as a result of coercion or fear of violence [27].

The association between male controlling behavior and the practice of contraception is similar to that obtained in Nigeria after analysis of their 2008 DHS data. In the study, women whose husbands had a controlling behaviour had reduced odds of practicing contraception compared to women whose husbands did not have a controlling behaviour [28]. This is because such men tend to restrict their spouses from seeking family planning services with the excuse that it encourages women to be unfaithful to their husbands [29].

The higher odds of practicing contraception among males who did not want their first child at delivery could be explained by avoidance of potential stresses experienced in the past to cater for the mother during pregnancy and the child. This means that such men will most likely practice contraception to prevent future unwanted births. The higher odds of practicing contraception among respondents who had more than one main sexual partner compared to those with just one main sexual partner is consistent with many studies in Africa [30, 31]. People with multiple sexual partners practice contraception, especially barrier methods, to limit or prevent pregnancies and sexually transmitted infections [31, 32].

### Conclusion and recommendations

This study points out that approximately one out of every four men practices contraception in the four districts of the Central Region of Ghana. Also, the educational level, perpetration of IPV, controlling behavior, number of main sexual partners and whether the first child was wanted are some male-specific characteristics that can predict the practice of contraception. Given these findings, there is a need for various stakeholders in the health sector to organize health campaigns aimed at educating men on the importance of contraception. Also, there is a need for multisectoral collaboration to discourage behaviors like male controlling behavior that hinders family planning services’ uptake. Finally, the Ghana Health Service must devise and implement strategies to render male-friendly services to involve men in family services.

### Study limitations

This study may have underestimated the prevalence of contraception among men mainly due to the possibility of covert usage of some modern methods by their partners. This study could also not report on specific forms of contraceptives used by men and or their partners. However, the evidence provided by this study can inform policymakers and program designers to ensure the successful design and implementation of family planning programs.

## Data Availability

The data supporting this study’s findings are openly available in MEDAT Data Repository – SAMRC at http://medat.samrc.ac.za/index.php/catalog/WW, reference Ghana (Baseline_men).

## Abbreviations

AOR: Adjusted Odds Ratio
BMGF: Bill and Melinda Gates Foundation
CI: Confidence Interval
COMBAT: Community-Based Action Teams
DHS: Demographic and Health Survey
GHS: Ghana Health Service
GSS: Ghana Statistical Service
ICF: (*originally* Inner City Fund)
IPV: Intimate Partner Violence
RRS: Rural Response System
SD: Standard Deviation
UOR: Unadjusted Odds Ratio
WHO: World Health Organization
